# Cannabis Use Frequency and Cannabis-Related Consequences in High-Risk Young Adults Across Cannabis Legalization

**DOI:** 10.1001/jamanetworkopen.2023.36035

**Published:** 2023-09-27

**Authors:** Amanda Doggett, Kyla Belisario, André J. McDonald, Mark A. Ferro, James G. Murphy, James MacKillop

**Affiliations:** 1 Peter Boris Centre for Addictions Research/Michael G. DeGroote Centre for Medicinal Cannabis Research, McMaster University/St. Joseph’s Healthcare, Hamilton, Ontario, Canada; 2 School of Public Health Sciences, University of Waterloo, Waterloo, Ontario, Canada; 3 Department of Psychology, The University of Memphis, Memphis, Tennessee

## Abstract

**Importance:**

A key concern about recreational cannabis legalization is increases in use and adverse consequences, particularly among young adults (aged 18-29 years) who have the highest prevalence of cannabis use, and especially in higher-risk, more vulnerable young adults. However, few longitudinal studies have examined patterns of cannabis consumption in high-risk young adults over the course of legalization.

**Objective:**

To examine changes in cannabis use frequency and cannabis-related consequences over recreational cannabis legalization in Canada in a longitudinal sample of high-risk young adults.

**Design, Setting, and Participants:**

Longitudinal observational cohort study following young adults in Ontario, Canada, aged 19.5 to 23.0 years who reported regular heavy episodic drinking (65% past-month cannabis use) at enrollment. Participants were surveyed every 4 months for 3 years between February 2017 and February 2020 (3 prelegalization waves, 4 postlegalization waves). Data were analyzed from March to May 2023.

**Exposures:**

Recreational cannabis legalization in Canada and 4 potential moderators of change: sex, income, education, and prelegalization cannabis use frequency.

**Main Outcomes and Measures:**

Cannabis use frequency and cannabis-related adverse consequences.

**Results:**

In a cohort of 619 high-risk young adults (baseline mean [SD] age, 21.0 [1.2] years; 346 female participants [55.9%]), omnibus model testing revealed significant overall decreases in both cannabis use frequency (*F* = 2.27_6, 3000.96_; *P* = .03) and cannabis-related consequences (*F* = 10.43_6, 3002.21_; *P* < .001) over time, but these changes were substantially moderated by prelegalization frequency (frequency: *F* = 7.52_24, 3021.88_; *P* < .001; consequences: *F* = 7.24_24, 2986.98_; *P* < .001). Follow-up tests showed individuals who used cannabis more frequently prelegalization significantly decreased their use and cannabis-related consequences postlegalization. In contrast, individuals who did not use cannabis prelegalization exhibited a small magnitude increase in frequency over time but nonsignificant changes in cannabis-related consequences. Sex, income, and education did not moderate changes over time.

**Conclusions and Relevance:**

In this cohort study of high-risk young adults, individuals using cannabis frequently prelegalization showed significant reductions in use and consequences over time, reflecting an aging out pattern. Small increases in use among participants with no prelegalization use were observed over time, but without parallel changes in cannabis-related consequences. The results did not reveal substantive adverse near-term outcomes across the legalization period, although a within-participants design cannot rule out the possibility of alternative trajectories in the absence of legalization.

## Key Points

**Question:**

Did recreational cannabis legalization in Canada change cannabis use frequency or cannabis-related consequences in high-risk young adults over time?

**Findings:**

In a longitudinal cohort study of 619 high-risk young adults in Ontario, Canada, there was an average decrease in cannabis use frequency and consequences across legalization, but patterns of use frequency and consequences over time differed significantly according to prelegalization use of cannabis.

**Meaning:**

These findings suggest that across Canadian cannabis legalization, high-risk young adults showed different patterns of change; those using cannabis frequently prelegalization exhibited reductions consistent with aging out, and those not using cannabis prelegalization exhibited modest increases in use over time.

## Teaser

This cohort study evaluates changes in cannabis use frequency and cannabis-related consequences over recreational cannabis legalization in Canada in a longitudinal sample of high-risk young adults.

## Introduction

A substantial liberalization of cannabis regulatory policy has taken place over the past decade in North America, including Canadian federal legalization of recreational (nonmedical) cannabis use for adults in October 2018. Although some differences exist by province, recreational legalization at the federal level in Canada provides a strict system for controlling the production, distribution, sale, and possession of cannabis.^1^ Notable components of this system include a federal age limit of 18 years for purchase (provincial limits range from 18-21 years), an individual possession limit of 30 g of dried cannabis or equivalent amounts of nondried cannabis, and strict regulations surrounding permissible packaging and marketing for cannabis products, specifically restriction of products that may be appealing to youths.^1^ Although original legislation only pertained to dried flower products, edible cannabis and concentrate products were allowed 1 year later in October 2019.^1^

In all jurisdictions where cannabis legalization takes place, a key concern has been that cannabis use and related harms would increase among youths and young adults due to easier access, growing social acceptability, declining perception of harm, lower prices, a wider array of products and modes of use, and increasing product potency.^2,3,4,5,6^ Young adults are of particularly high priority because they have the highest prevalence of use. In Canada, for example, cannabis use is the second most prevalent substance used for those aged 20 to 24 years (33% consumed in the past year),^7^ after alcohol. Moreover, cannabis and alcohol use tend to occur concurrently in this group and co-use is associated with additional adverse consequences.^8,9^ Young adults also have the highest prevalence of cannabis use disorder.^10^ Finally, a primary concern is that cannabis use in young adults may interfere with psychosocial development, such as academic and vocational attainment, and neurodevelopment, which is estimated to continue until approximately 25 years of age.^11,12^ Accordingly, young adults, particularly those already using cannabis and/or alcohol, represent a high-risk group warranting surveillance in the context of legalization.

Evidence examining the impacts of legalization on young adults from US jurisdictions is generally mixed. One study focusing on postsecondary students in Oregon found an increase in cannabis use after recreational cannabis legalization,^13^ but a broader study found no significant changes in young adult cannabis use across legalization in Colorado, Washington, Alaska, or Oregon.^14^ In Canada, evidence is sparse regarding the impact of legalization on cannabis use and cannabis-related consequences. Prelegalization data from the 2018 National Cannabis Survey indicated that the prevalence of cannabis use in the past 3 months among those aged 18 to 24 years was 28%, the highest prevalence of all age groups.^15^ Although some studies have found that cannabis use (and frequency of use) increased among youths and young adults following legalization, most studies have not found a pronounced or statistically significant increase.^3,16,17^ Conversely, evidence suggestions that there have been some impacts on public health more broadly, including increases in incidence of youth hospitalizations and emergency department visits related to cannabis use and increased cannabis-related traffic fatalities.^17,18,19^

Although young adults consume cannabis at a higher prevalence than other age groups, there is a dearth of research focusing on this population. Moreover, there have been few longitudinal studies examining the impact of legalization, which represents a substantial research gap. Longitudinal data provide richer information than repeated cross-sectional surveys, allowing for characterization of within-person changes, the comparison of trajectories of cannabis use, estimation of rates of change, and moderators of changes, such as prelegalization cannabis use status, sex, or income.^18,20^ The purpose of this study was to address these research gaps by examining the association of cannabis legalization with cannabis use frequency and cannabis-related consequences using longitudinal data from a cohort of young adults who already used substances prelegalization. These individuals represent a high-risk subgroup of the population given their age and their existing propensity to engage in substance use. The first aim of this study was to examine the overall changes in cannabis use outcomes, namely frequency of use and negative consequences from cannabis, both descriptively and analytically. The second aim was to examine credible moderators of differences in changes over time, namely sex, income, education, and prelegalization frequency of cannabis use.

## Methods

### Design and Participants

This study used a longitudinal cohort design, using data from an existing cohort of high-risk young adults in Ontario, Canada. Inclusion criteria were as follows: aged 19.5 to 23.0 years at first data collection; engaged in 2 or more heavy episodic drinking episodes; fluency in written English; and no current or past experiences in psychosis. Substance use criteria were intended to recruit a sample with epidemiologically common patterns of substance use associated with elevated risk for adverse consequences in young adults. This cohort study was reviewed and approved by the Hamilton integrated research ethics board. Informed consent was obtained from participants in-person during their baseline visit. This study followed the Strengthening the Reporting of Observational Studies in Epidemiology (STROBE) guidelines.

Participant enrollment started in April 2017 on a rolling basis, with follow-up assessments administered every 4 months. To assess changes in cannabis consumption frequency and related consequences before and after legalization, assessments were rebinned in 4-month periods to align with legalization in Canada (October 17, 2018). In total, 7 time points were included in the analysis: 2 prelegalization time points (April 17, 2017, to February 16, 2018, and February 17 to June 16, 2018), 3 postlegalization time points (February 17 to June 16, 2019; June 17 to October 16, 2019; and October 17, 2019, to February 16, 2020), and 2 transition periods between the prelegalization and postlegalization periods (June 17 to October 16, 2018, and October 17, 2018, to February 16, 2019). A visualization of participation over these time points is provided in eFigure 1 in Supplement 1. The first time point was longer than subsequent time points because of the rolling recruitment process. The first transition period coincides with the passing of the Cannabis Act in June 2018 and the second reflects the period immediately following legalization.

Participants were required to have at least 1 prelegalization and postlegalization observation assessment and have demonstrated adequate attention on the majority of quality control items to be included in the current study. Of the original cohort, 85% met this criterion. An attrition analysis examining the removed individuals is available in the eTable in Supplement 1; those excluded were more likely to be male and had moderately higher average cannabis use frequency and Brief Marijuana Consequences Questionnaire (B-MACQ) scores.

### Measures

Participants were assessed for lifetime cannabis use and, among those who had used cannabis, cannabis frequency during the past month was assessed using the Alcohol, Smoking, Substance Involvement Screening Test, with response options of none, monthly, weekly, daily, or multiple times daily.^21^ Using response options from these 2 questions, the frequency categories examined in this study were never use (ie, those who have never used cannabis in their life), no (current) use, occasional use (monthly), regular use (weekly), and frequent use (daily or multiple times daily). Consequences related to cannabis use were measured for the past 4 months using the B-MACQ,^22^ which creates a sum score of number of problems endorsed (0 to 21). To visualize B-MACQ scores, groups of 0 consequences, 1 to 4 consequences, and 5 or more consequences were created, but in analyses this measure was treated as continuous. The Cannabis Use Disorder Identification Test (CUDIT), an 8-item clinical screening tool for cannabis misuse^23^ was administered at baseline to characterize clinical severity; a cutoff of 6 has been validated in young adults.^24^ To contextualize the sample with a clinical cutoff, frequency and B-MACQ in relation to the CUDIT are shown in eFigure 2 in Supplement 1. Collectively, the separate outcomes for frequency and consequences show high overlap with the CUDIT cutoff at baseline.

### Statistical Analysis

All statistical analyses and data visualizations were conducted in R version 4.2.2 (R Project for Statistical Computing). Descriptive analyses used alluvial plots and additional data visualization to depict changes across legalization. To efficiently summarize available data, descriptive visualizations show transitions between highest use category over the prelegalization and postlegalization time points. In statistical analyses, cannabis use frequency and consequences associated with cannabis use were modeled as outcomes across all 7 time points using linear mixed effects models (LMMs). The use of LMMs accounted for the cross-linked longitudinal nature of the data, capturing individual-level variation. Although outcome data were not continuous by nature, LMMs are robust to departures from distributional assumptions of normality,^25^ and numerous studies have highlighted that ordinal or scale-type data can be appropriately treated as continuous.^26,27,28^ Examination of residual plots and sensitivity analyses using a robust scoring equations estimator further validated the linear approximation of the outcome data. All models controlled for age, sex, income, and education. For aim 1, analyses examined the outcomes of time on overall cannabis use frequency and consequences. For aim 2, interaction effects were added to examine the potential moderating effects of (1) sex assigned at birth; (2) median household income; (3) minimum 4-year undergraduate degree; and (4) maximum prelegalization frequency of use (at time point 1 or 2). Income and education were assessed at follow-up rather than baseline due to the young age of this cohort at enrollment. Significant omnibus results of time and the interaction of time with moderators were followed up using post-hoc pairwise contrasts to examine differences between time points. Emphasis was on whether transition or postlegalization time points were significantly different from both prelegalization time points. Statistical tests were considered significant at α = .05. Data were analyzed from March to May 2023.

## Results

### Sample Characteristics

Table 1 presents descriptive statistics for this study sample of 619 participants. The mean (SD) age was 21.0 (1.2) years, 346 (56%) were female, and 330 (53%) had a bachelor’s degree at the most recently available postlegalization time point. The sample sizes at each wave for the final sample were 418 at time 1, 578 at time 2, 530 at time 3, 509 at time 4, 524 at time 5, 531 at time 6, and 529 at time 7. Prelegalization, occasional cannabis use was most common (206 participants [33%]). Among those who used cannabis, mean (SD) prelegalization experiences of cannabis-related consequences in the past month was 2.53 (3.91), with a moderate spread across participants.

**Table 1.** Sample Descriptive Statistics

Characteristic	Participants, No. (%) (N = 619)
Sex^a^	
Female	346 (55.9)
Male	273 (44.1)
Age, mean (SD), y	21.43 (1.19)
Household income^b^	
<$15 000	69 (11.1)
$15 000-$29 999	113 (18.3)
$30 000-$44 999	76 (12.3)
$45 000-$59 999	63 (10.2)
$60 000-$74 999	61 (9.9)
$75 000-$89 999	48 (7.8)
$90 000-$104 999	51 (8.2)
$105 000-$119 999	38 (6.1)
≥$120 000	100 (16.2)
Bachelor’s degree or higher^b^	330 (53.3)
Prelegalization cannabis use frequency, mean (SD)	
Mean (SD)	1.17 (1.17)
Never use	77 (12.4)
No current use	140 (22.6)
Occasional use	206 (33.3)
Regular use	106 (17.1)
Frequent use	90 (14.5)
Prelegalization B-MACQ score, mean (SD)^c^	2.53 (3.91)
CUDIT score, mean (SD)^d^	4.77 (6.07)

Abbreviations: B-MACQ, Brief Marijuana Consequences Questionnaire; CUDIT, Cannabis Use Disorder Identification Test.

^a^ Gender and sex overlapped 99.7% in this sample.

^b^ From most recently available postlegalization time point.

^c^ Scored from 0 to 21. The higher the score, the more consequences an individual experienced from marijuana use.

^d^ Scored from 0 to 32. The higher the score, the more likely an individual is to have cannabis use disorder.

### Overall Changes Across Legalization

**Figure 1.**
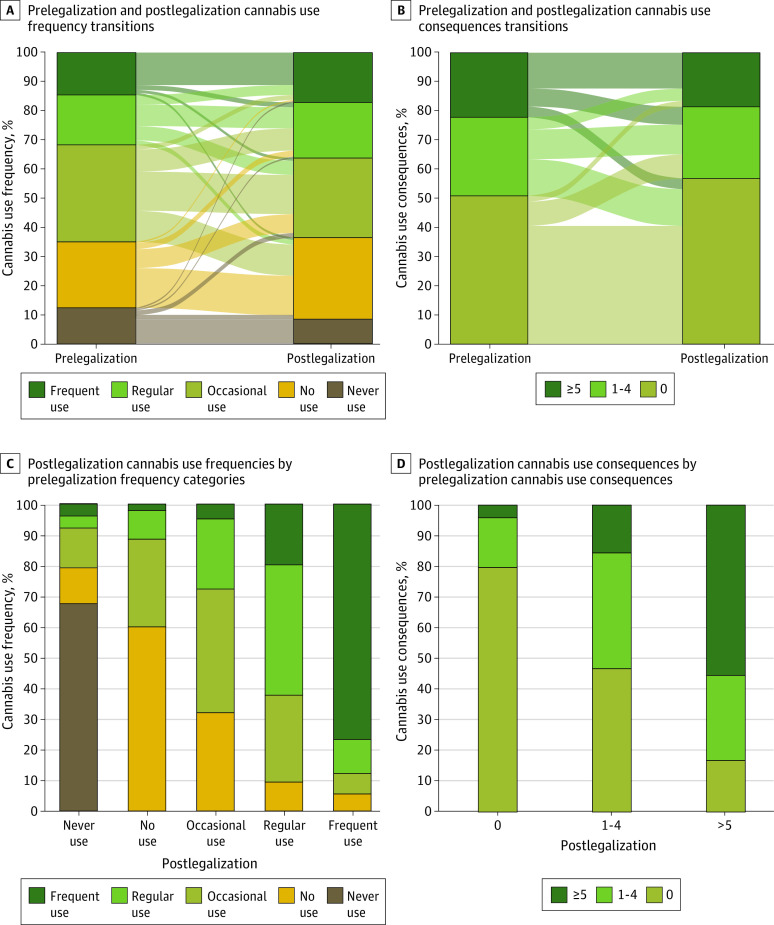
Prelegalization and Postlegalization Transitions of Cannabis Use Frequency and Consequences Cannabis use frequency and consequences are summarized in this figure by an individual’s greatest value during prelegalization (times 1-2) and postlegalization (times 4-7) time points.

Figure 1 descriptively demonstrates the shifts in cannabis use frequency and B-MACQ scores over time. Although occasional cannabis use was the largest category prelegalization, no use was the largest category postlegalization. Occasional cannabis use was the most dynamic category, as only 83 of those who started with occasional use prelegalization (40%) also reported occasional use post legalization; 66 transitioned to no use (32%), and 47 increased to regular use (23%). A B-MACQ score of 0 (no cannabis-related consequences) was most prevalent prelegalization and postlegalization; however, this category grew between these time points (315 participants [51%] and 352 participants [57%], respectively); this was the most stable category over time. The majority of those who reported 1 to 4 consequences over time transitioned to either no consequences (78 participants [47%]) or 5 or more consequences (26 participants [16%]) postlegalization. Those in the frequent cannabis use category generally maintained high levels of cannabis use and consequences over time; 69 of those using frequently prelegalization (77%) remained in that category postlegalization, and 76 of those with a B-MACQ score of 5 or more prelegalization (56%) were still experiencing 5 or more cannabis-related consequences postlegalization.

**Figure 2.**
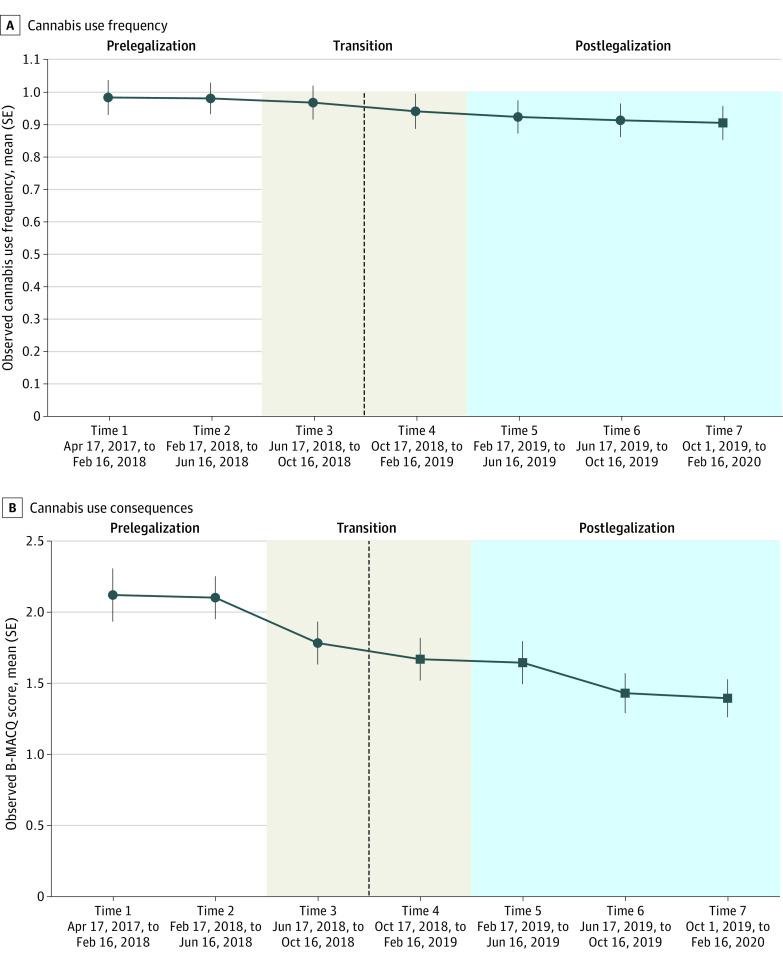
Overall Changes in Cannabis Use and Consequences Over 7 Waves The dotted line indicates legalization of cannabis in Canada on October 17, 2018. Square time points indicate *P* < .05 vs time 1 and 2.

Omnibus testing of LMM main effect models showed that cannabis use frequency (*F* = 2.27_6, 3000.96_; *P* = .03) and cannabis consequences (*F* = 10.43_6, 3002.21_; *P* < .001) changed significantly over time. Pairwise contrasts examined which time points were significantly different than prelegalization time points, and are presented in Table 2. Figure 2 illustrates the observed changes in mean cannabis use frequency and consequences over time; significant differences from prelegalization time points determined through pairwise contrasts are also identified. Mean cannabis use frequency decreased modestly over time, such that use at time 7 was significantly lower than that of time 1 (β = 0.152; 95% CI, 0.027-0.277; *P* = .006). Cannabis use consequences overall also decreased over time, such that time points 4 through 7 (ie, postlegalization) were significantly lower than both prelegalization time points, and time point 3 was significantly lower than prelegalization time point 1.

**Table 2.** Post Hoc Pairwise Contrasts Comparing Prelegalization Time Points to All Other Time Points

Time periods	Cannabis use frequency by time point				Cannabis use consequences by time point			
	1^a^		2^b^		1^a^		2^b^	
**β (95% CI)**	***P* value**	**β (95% CI)**	***P* value**	**β (95% CI)**	***P* value**	**β (95% CI)**	***P* value**
Overall effect								
2	0.081 (−0.040 to 0.204)	.45	NA	NA	0.191 (−0.198 to 0.580)	.77	NA	NA
3	0.079 (−0.046 to 0.204)	.51	−0.002 (−0.116 to 0.111)	>.99	0.451 (0.055 to 0.847)	.01	0.259 (−0.100 to 0.619)	.34
4	0.092 (−0.034 to 0.219)	.32	0.012 (−0.103 to 0.126)	>.99	0.593 (0.191 to 0.995)	<.001	0.402 (0.038 to 0.765)	.02
5	0.102 (−0.023 to 0.226)	.20	0.021 (−0.093 to 0.135)	>.99	0.592 (0.197 to 0.987)	<.001	0.400 (0.040 to 0.761)	.02
6	0.102 (−0.023 to 0.227)	.20	0.021 (−0.092 to 0.134)	>.99	0.735 (0.339 to 1.131)	<.001	0.544 (0.185 to 0.903)	<.001
7	0.152 (0.027 to 0.277)	.006	0.071 (−0.042 to 0.185)	.51	0.842 (0.446 to 1.238)	<.001	0.651 (0.291 to 1.010)	<.001
Prelegalization use moderated effects								
Never use								
2	−0.007 (−0.335 to 0.321)	>.99	NA	NA	−0.007 (−1.051 to 1.037)	>.99	NA	NA
3	−0.064 (−0.395 to 0.267)	>.99	−0.057 (−0.36 to 0.247)	>.99	−0.061 (−1.114 to 0.992)	>.99	−0.054 (−1.018 to 0.910)	>.99
4	−0.137 (−0.478 to 0.203)	.90	−0.131 (−0.442 to 0.181)	.88	−0.145 (−1.228 to 0.938)	>.99	−0.138 (−1.127 to 0.851)	>.99
5	−0.227 (−0.563 to 0.108)	.42	−0.221 (−0.531 to 0.090)	.35	−0.138 (−1.205 to 0.929)	>.99	−0.131 (−1.117 to 0.855)	>.99
6	−0.146 (−0.475 to 0.182)	.85	−0.140 (−0.441 to 0.162)	.82	−0.089 (−1.134 to 0.956)	>.99	−0.082 (−1.038 to 0.875)	>.99
7	−0.252 (−0.584 to 0.081)	.28	−0.245 (−0.549 to 0.060)	.21	−0.177 (−1.236 to 0.882)	>.99	−0.170 (−1.138 to 0.799)	>.99
No use								
2	−0.005 (−0.256 to 0.245)	>.99	NA	NA	−0.053 (−0.850 to 0.744)		NA	NA
3	−0.261 (−0.515 to −0.006)	.04	−0.255 (−0.486 to −0.025)	.02	−0.354 (−1.163 to 0.455)	.86	−0.301 (−1.033 to 0.431)	.89
4	−0.262 (−0.516 to −0.009)	.04	−0.257 (−0.487 to −0.027)	.02	−0.426 (−1.234 to 0.381)	.71	−0.373 (−1.104 to 0.358)	.74
5	−0.336 (−0.59 to −0.081)	.002	−0.330 (−0.563 to −0.097)	<.001	−0.593 (−1.402 to 0.216)	.32	−0.540 (−1.280 to 0.200)	.32
6	−0.336 (−0.588 to −0.083)	.002	−0.330 (−0.559 to −0.101)	<.001	−0.617 (−1.422 to 0.187)	.26	−0.564 (−1.292 to 0.164)	.25
7	−0.311 (−0.566 to −0.056)	.006	−0.306 (−0.537 to −0.074)	.002	−0.444 (−1.255 to 0.367)	.67	−0.391 (−1.126 to 0.345)	.70
Occasional use								
2	0.174 (−0.030 to 0.378)	.15	NA	NA	0.024 (−0.627 to 0.674)	>.99	NA	NA
3	0.211 (0.004 to 0.419)	.04	0.037 (−0.156 to 0.230)	>.99	0.464 (−0.198 to 1.125)	.37	0.440 (−0.173 to 1.053)	.34
4	0.218 (0.009 to 0.427)	.03	0.043 (−0.151 to 0.237)	>.99	0.538 (−0.129 to 1.205)	.21	0.514 (−0.102 to 1.131)	.17
5	0.190 (−0.015 to 0.395)	.09	0.016 (−0.175 to 0.206)	>.99	0.598 (−0.055 to 1.251)	.10	0.575 (−0.032 to 1.181)	.08
6	0.149 (−0.057 to 0.355)	.33	−0.025 (−0.217 to 0.166)	>.99	0.451 (−0.206 to 1.107)	.40	0.427 (−0.181 to 1.034)	.37
7	0.170 (−0.038 to 0.377)	.19	−0.005 (−0.197 to 0.188)	>.99	0.527 (−0.134 to 1.189)	.22	0.504 (−0.108 to 1.116)	.19
Regular use								
2	0.149 (−0.142 to 0.440)	.74	NA	NA	0.795 (−0.132 to 1.721)	.15	NA	NA
3	0.172 (−0.124 to 0.468)	.61	0.023 (−0.242 to 0.287)	>.99	1.306 (0.361 to 2.251)	<.001	0.511 (−0.332 to 1.355)	.56
4	0.303 (−0.008 to 0.614)	.06	0.154 (−0.124 to 0.431)	.66	1.370 (0.378 to 2.361)	<.001	0.575 (−0.308 to 1.459)	.47
5	0.407 (0.108 to 0.707)	.001	0.258 (−0.010 to 0.526)	.07	1.457 (0.503 to 2.411)	<.001	0.663 (−0.190 to 1.515)	.25
6	0.393 (0.091 to 0.695)	.002	0.244 (−0.026 to 0.514)	.11	2.150 (1.187 to 3.112)	<.001	1.355 (0.498 to 2.212)	<.001
7	0.539 (0.239 to 0.839)	<.001	0.390 (0.122 to 0.657)	<.001	2.204 (1.246 to 3.162)	<.001	1.409 (0.556 to 2.263)	<.001
Frequent use								
2	−0.007 (−0.331 to 0.317)	>.99	NA	NA	0.684 (−0.355 to 1.723)	.45	NA	NA
3	0.287 (−0.049 to 0.622)	.15	0.294 (−0.004 to 0.591)	.06	1.300 (0.225 to 2.375)	.007	0.616 (−0.330 to 1.562)	.47
4	0.312 (−0.022 to 0.646)	.09	0.319 (0.024 to 0.613)	.02	2.296 (1.221 to 3.371)	<.001	1.612 (0.671 to 2.553)	<.001
5	0.538 (0.206 to 0.870)	<.001	0.545 (0.249 to 0.841)	<.001	2.290 (1.226 to 3.355)	<.001	1.606 (0.665 to 2.547)	<.001
6	0.559 (0.219 to 0.898)	<.001	0.566 (0.264 to 0.867)	<.001	2.968 (1.879 to 4.056)	<.001	2.284 (1.324 to 3.243)	<.001
7	0.737 (0.409 to 1.066)	<.001	0.744 (0.453 to 1.035)	<.001	3.193 (2.140 to 4.245)	<.001	2.508 (1.583 to 3.434)	<.001

Abbreviation: NA, not applicable.

^a^ Time point 1 was April 17, 2017 to February 16, 2018.

^b^ Time point 2 was February 17, 2018 to June 16, 2018.

### Moderators of Changes Over Time

**Figure 3.**
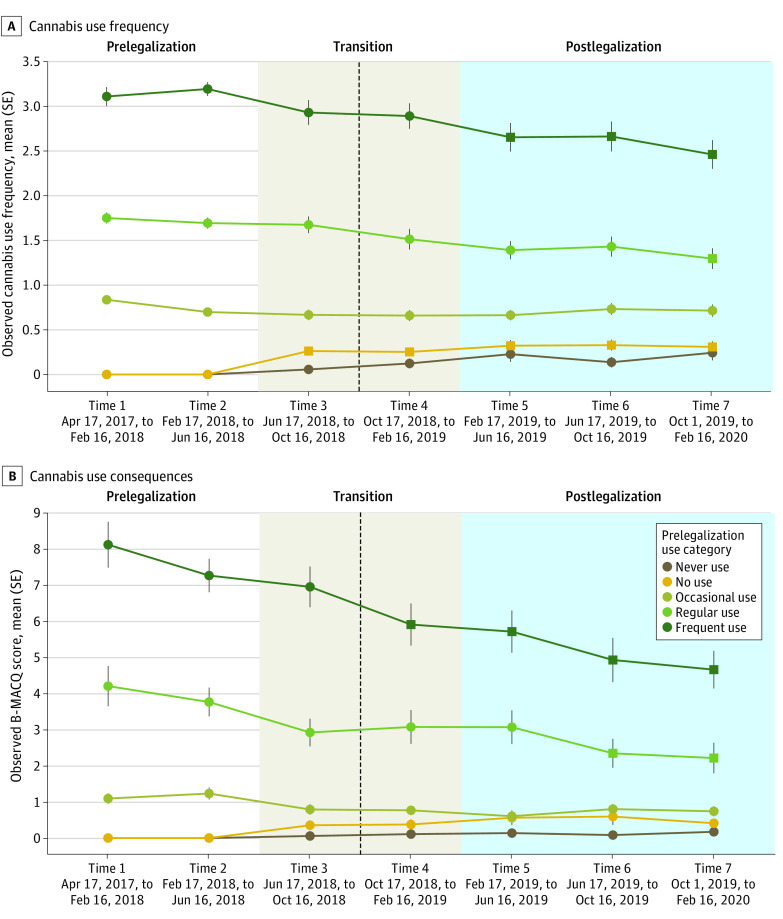
Changes in Cannabis Use and Consequences Over 7 Waves According to Prelegalization Cannabis Use Frequency The dotted line indicates legalization of cannabis in Canada on October 17, 2018. Square time points indicate *P* < .05 vs time 1 and 2.

Omnibus testing of LMMs examining interactions with time demonstrated that prelegalization use frequency significantly moderated the outcome of time for both cannabis use frequency (*F* = 7.52_24, 3021.88_; *P* < .001) and cannabis use consequences (*F* = 7.24_24, 2986.98_; *P* < .001). Pairwise contrasts for these interaction models are also presented in Table 2. Figure 3 shows observed means by prelegalization use category, and the significant interaction effects determined through pairwise contrasts are also identified. Only those who reported frequent cannabis use or no cannabis use prelegalization showed significant changes in use over time. Participants who initially reported frequent prelegalization use had significantly lower mean cannabis use frequency from times 5 to 7 and had significantly lower cannabis use consequences at times 4 to 7, compared with both prelegalization time points. Those who initially reported no use showed significantly higher mean cannabis use frequency between time points 3 to 7 compared with prelegalization but did not exhibit increases in negative cannabis consequences.

Omnibus testing of other interaction effects revealed no moderating effects of sex (*F* = 0.89_6, 3003.52_; *P* = .50), income (*F* = 0.92_6, 3002.31_; *P* = .48), or education (*F* = 0.69_6, 3002.36_; *P* = .66) on cannabis use frequency. Similarly, there were no moderating effects of sex (*F* = 0.22_6, 2999.21_; *P* = .97), income (*F* = 1.05_6, 2997.87_; *P* = .39), or education (*F* = 1.45_6, 2997.72_; *P* = .19) on cannabis use consequences.

## Discussion

This study examined changes in cannabis use and consequences following recreational cannabis legalization in Canada in a sample of high-risk young adults, addressing the common concern that legalization may precipitate increases in use, particularly in this age group. Rather than detecting increases, however, the results revealed decreases overall, which is broadly consistent with substance use trajectories that might be expected among this age group in the absence of any policy change. Correspondingly, the changes observed in this study did not appear to be markedly changed by cannabis legalization.

Important moderating effects were present according to prelegalization cannabis frequency status. Individuals using cannabis most frequently prelegalization exhibited, on average, a significant reduction in use postlegalization. Correspondingly, this group also exhibited a significant reduction in cannabis-related consequences. In contrast, participants who were not recent cannabis users prelegalization increased their frequency of use over time, on average. Notably, this increase was modest and began at time 3 (prior to legalization), and this group exhibited no significant changes in cannabis-related consequences. In other words, although frequency increased for this subgroup, such an increase did not lead to problematic outcomes during the study period. Also of note, participants who had never used cannabis prior to legalization exhibited no significant increases in use or consequences postlegalization.

Among the prelegalization frequent use group, the observed changes are consistent with developmentally normative declines in this age group over time, sometimes referred to as aging out^29^ of substance misuse; previous studies have also indicated similar findings in youth samples.^3^ A caveat, of course, is that it was not possible in this study to conclude that legalization did not attenuate or otherwise alter this aging-out trend. It should also be noted that average potency of cannabis products has generally increased over time,^30,31^ and increases in product potency can lead to decreases in use frequency, although this would likely be a modest effect over the study period and we might expect a parallel effect in cannabis-related consequences. For the prelegalization nonuse (but not never-use) group, the modest increases observed are consistent with a study of community adults that examined changes in cannabis use frequency 1-year after legalization.^20^ Notably, while individuals in this study appeared to reinitiate cannabis use at a modest level, given that this began prior to legalization, it is unlikely that the policy change was a specific precipitant. It should also be noted that although these trends tended to reflect aging out and are generally consistent with other literature, results could also reflect regression to the mean, in particular because this cohort was a subset of the young adult population who engaged in higher risk substance use at baseline. Nonetheless, given that some of these participants did exhibit notable increases in frequency alongside the fact that a majority of the those using cannabis at the highest frequency did not reduce their use over time, the determinants of escalation and persistence of high frequency cannabis use in young adults warrants further examination.

This study aligns with research from US jurisdictions which have largely found that legalization has not drastically altered consumption patterns among youths and young adults.^32^ In the Canadian context, existing legalization studies are sparse and methodologically distinct from the present one, making comparison difficult. For example, a frequently referenced study^15^ reported increases in cannabis use frequency over legalization, but was largely descriptive in nature. Another concluded that cannabis legalization increased use among adults, but dichotomized prelegalization and postlegalization periods as opposed to examining trends between time points.^33^ Both aforementioned studies used repeat cross-sectional designs and focused on the general adult population. This further demonstrates a benefit of the present study; the longitudinal design allowed for the examination of within-person changes, which is critical to understand how people change following policy implementation, and has been highlighted as a priority for evaluating cannabis legalization.^18^ Moreover, the focus on high-risk young adults prelegalization was an important feature as they would be expected to be particularly vulnerable in the new policy climate.

In part, the lack of considerable change over such a large-scale policy shift could be because cannabis use in Canada was already quite normalized prelegalization.^34^ Prelegalization perceptions among Canadians who used cannabis were that it was already fairly easy to access,^35^ and risk perceptions were generally low.^36^ As such, any changes in access or social acceptability brought on by cannabis legalization may have been fairly inconsequential on individual use patterns in this age group. That said, in the broader context of population-level changes, cannabis legalization was fairly recent and continued population surveillance is needed to further understand any impacts.

Beyond prelegalization cannabis use, no moderation of changes was present according to sex, income, or education. This is generally favorable for public health insofar as it does not suggest health disparities for these common demographic variables related to impacts of cannabis legalization in young adults.

### Strengths and Limitations

The findings of this study should be considered in the context of its strengths and limitations. To our knowledge, this is the first evaluation of Canadian cannabis legalization among a young adult population that used a robust longitudinal approach. Specifically, these data consisted of multiple linked prelegalization and postlegalization time points, which is a rare feature as many studies rely on repeated cross-sectional designs or only focus on postlegalization patterns. Given these key elements, this study is well-positioned to inform cannabis legalization in other jurisdictions, with the understanding that implementation of cannabis legalization policies can vary greatly, and results should be considered alongside the larger body of literature and region-specific data. Important considerations include that the present study reports findings from 1 nonprobability sample of young adults, which is not necessarily nationally representative of this age group across Canada. Furthermore, this study focused on individuals who were engaged in substance use prelegalization, as opposed to the general population. High-risk individuals are arguably of greatest interest for evaluating legalization effects, serving as a early indicator of changes in use, but the results should be interpreted with the caveat that cannabis legalization may have changed patterns of use and consequences in other groups not captured by this study sample.

At a broader level, a limitation of these findings is the lack of a control group to evaluate cannabis legalization as a natural experiment more fully. Although we principally observed decreases in use and problems, it is still possible that legalization attenuated or otherwise altered the patterns we might have observed in absence of this policy change, and future research may consider matching available Canadian data to an appropriate control jurisdiction to examine legalization as a natural experiment.^18,37^ This study also focused only on the initial policy change legalizing dried flower products; the legalization of cannabis edibles and concentrate products could have differential impacts not captured in this study. Attrition analyses indicated that those excluded from the study had modestly higher average cannabis use frequency than those included, which may have reduced our ability to model legalization on those who engaged in heavier cannabis use. However, this concern is partially mitigated by the generally high retention rate among participants, many of whom did engage in persistently frequent cannabis use. Additionally, although we had 4 postlegalization follow-ups, legalization may have long-term health and social impacts that are not detectable during the time window that we studied, highlighting the need for continued monitoring. These considerations notwithstanding, this study adds to the limited longitudinal literature evaluating the outcomes of recreational cannabis legalization.

## Conclusions

In this cohort study of high-risk young adults, findings revealed general decreases and indicated that those who used cannabis frequently prelegalization on average exhibited substantive decreases in use and cannabis-related consequences postlegalization, consistent with expected aging-out patterns of substance use. Conversely, those not using cannabis prelegalization showed a significant but modest increase in use over time. Given the continued trend toward cannabis legalization globally, further longitudinal surveillance is critical to evaluate the consequences of cannabis legalization empirically and promote evidence-informed public policy.

## Notes

Supplement 1. **eTable.** Attrition Analysis **eFigure 1.** Distribution of Participation Across 7 Study Waves **eFigure 2. **Prelegalization B-MACQ and CUDIT Summaries by Cannabis Use Category

Supplement 2.
Data Sharing Statement

## References

[1] Government of Canada. Cannabis Act. Federal Government of Canada; 2018.

[2] Hall W, Lynskey M. Evaluating the public health impacts of legalizing recreational cannabis use in the United States. Addiction. 2016;111(10):1764-1773. doi:10.1111/add.13428 27082374

[3] Haines-Saah RJ, Fischer B. Youth cannabis use and legalization in canada—reconsidering the fears, myths and facts three years in. J Can Acad Child Adolesc Psychiatry. 2021;30(3):191-196. 34381511 PMC8315217

[4] Kelsall D. Cannabis legislation fails to protect Canada’s youth. CMAJ. 2017;189(21):E737-E738. doi:10.1503/cmaj.170555 28554946 PMC5449235

[5] Doggett A, Battista K, Jiang Y, de Groh M, Leatherdale ST. Patterns of cannabis use among canadian youth over time; examining changes in mode and frequency using latent transition analysis. Subst Use Misuse. 2022;57(4):548-559. doi:10.1080/10826084.2021.2019785 34994289

[6] McDonald AJ, Roerecke M, Mann RE. Adolescent cannabis use and risk of mental health problems-the need for newer data. Addiction. 2019;114(10):1889-1890. doi:10.1111/add.14724 31256420

[7] Canadian Centre on Substance Use and Addiction. Canadian Drug Summary. Alcohol. 2019.

[8] Subbaraman MS, Kerr WC. Simultaneous versus concurrent use of alcohol and cannabis in the National Alcohol Survey. Alcohol Clin Exp Res. 2015;39(5):872-879. doi:10.1111/acer.12698 25872596 PMC4399000

[9] Meyer W, Leece P. Risk factors for simultaneous use of alcohol and cannabis. Public Health Ontario; 2018.

[10] Qadeer RA, Georgiades K, Boyle MH, Ferro MA. An epidemiological study of substance use disorders among emerging and young adults. Can J Psychiatry. 2019;64(5):313-322. doi:10.1177/0706743718792189 30071752 PMC6591883

[11] Grant CN, Bélanger RE. Cannabis and Canada’s children and youth. Paediatr Child Health. 2017;22(2):98-102. doi:10.1093/pch/pxx017 29480902 PMC5804770

[12] Lubman DI, Cheetham A, Yücel M. Cannabis and adolescent brain development. Pharmacol Ther. 2015;148:1-16. doi:10.1016/j.pharmthera.2014.11.009 25460036

[13] Kerr DCR, Bae H, Koval AL. Oregon recreational marijuana legalization: changes in undergraduates’ marijuana use rates from 2008 to 2016. Psychol Addict Behav. 2018;32(6):670-678. doi:10.1037/adb0000385 30010351

[14] Cerdá M, Mauro C, Hamilton A, . Association between recreational marijuana legalization in the United States and changes in marijuana use and cannabis use disorder from 2008 to 2016. JAMA Psychiatry. 2020;77(2):165-171. doi:10.1001/jamapsychiatry.2019.3254 31722000 PMC6865220

[15] Rotermann M. Looking-back-from-2020-how-cannabis-use-and-related-behaviours-changed-in-Canada. Heal Reports; 2021. 10.25318/82-003-x202100400001-eng 33881274

[16] Fischer B, Lee A, Robinson T, Hall W. An overview of select cannabis use and supply indicators pre- and post-legalization in Canada. Subst Abuse Treat Prev Policy. 2021;16(1):77. doi:10.1186/s13011-021-00405-7 34620191 PMC8496143

[17] Rubin-Kahana DS, Crépault J-F, Matheson J, Le Foll B. The impact of cannabis legalization for recreational purposes on youth: a narrative review of the Canadian experience. Front Psychiatry. 2022;13:984485. doi:10.3389/fpsyt.2022.984485 36213917 PMC9539831

[18] Athanassiou M, Dumais A, Zouaoui I, Potvin S. The clouded debate: a systematic review of comparative longitudinal studies examining the impact of recreational cannabis legalization on key public health outcomes. Front Psychiatry. 2023;13:1060656. doi:10.3389/fpsyt.2022.1060656 36713920 PMC9874703

[19] Farmer CM, Monfort SS, Woods AN. Changes in traffic crash rates after legalization of marijuana: results by crash severity. J Stud Alcohol Drugs. 2022;83(4):494-501. doi:10.15288/jsad.2022.83.494 35838426 PMC9318699

[20] Turna J, Belisario K, Balodis I, Van Ameringen M, Busse J, MacKillop J. Cannabis use and misuse in the year following recreational cannabis legalization in Canada: a longitudinal observational cohort study of community adults in Ontario. Drug Alcohol Depend. 2021;225(April):108781. doi:10.1016/j.drugalcdep.2021.108781 34098383

[21] WHO ASSIST Working Group. The Alcohol, Smoking and Substance Involvement Screening Test (ASSIST): development, reliability and feasibility. Addiction. 2002;97(9):1183-1194. doi:10.1046/j.1360-0443.2002.00185.x 12199834

[22] Simons JS, Dvorak RD, Merrill JE, Read JP. Dimensions and severity of marijuana consequences: development and validation of the Marijuana Consequences Questionnaire (MACQ). Addict Behav. 2012;37(5):613-621. doi:10.1016/j.addbeh.2012.01.008 22305645 PMC3307958

[23] Adamson SJ, Kay-Lambkin FJ, Baker AL, . An improved brief measure of cannabis misuse: the Cannabis Use Disorders Identification Test-Revised (CUDIT-R). Drug Alcohol Depend. 2010;110(1-2):137-143. doi:10.1016/j.drugalcdep.2010.02.017 20347232

[24] Schultz NR, Bassett DT, Messina BG, Correia CJ. Evaluation of the psychometric properties of the cannabis use disorders identification test—revised among college students. Addict Behav. 2019;95(February):11-15. doi:10.1016/j.addbeh.2019.02.016 30798191

[25] Schielzeth H, Dingemanse NJ, Nakagawa S, . Robustness of linear mixed-effects models to violations of distributional assumptions. Methods Ecol Evol. 2020;11(9):1141-1152. doi:10.1111/2041-210X.13434

[26] Carifio J, Perla R. Resolving the 50-year debate around using and misusing Likert scales. Med Educ. 2008;42(12):1150-1152. doi:10.1111/j.1365-2923.2008.03172.x 19120943

[27] Norman G. Likert scales, levels of measurement and the “laws” of statistics. Adv Health Sci Educ Theory Pract. 2010;15(5):625-632. doi:10.1007/s10459-010-9222-y 20146096

[28] Sullivan GM, Artino AR Jr. Analyzing and interpreting data from likert-type scales. J Grad Med Educ. 2013;5(4):541-542. doi:10.4300/JGME-5-4-18 24454995 PMC3886444

[29] Cristiano N, Sharif-Razi M. Marijuana, cocaine, and ecstasy in this day and age: a quantitative investigation of the “aging out” process. J Subst Use. 2019;24(4):400-406. doi:10.1080/14659891.2019.1581288

[30] Chandra S, Radwan MM, Majumdar CG, Church JC, Freeman TP, ElSohly MA. New trends in cannabis potency in USA and Europe during the last decade (2008-2017). Eur Arch Psychiatry Clin Neurosci. 2019;269(1):5-15. doi:10.1007/s00406-019-00983-5 30671616

[31] Mahamad S, Wadsworth E, Rynard V, Goodman S, Hammond D. Availability, retail price and potency of legal and illegal cannabis in Canada after recreational cannabis legalisation. Drug Alcohol Rev. 2020;39(4):337-346. doi:10.1111/dar.13069 32291811

[32] Hall W, Lynskey M. Assessing the public health impacts of legalizing recreational cannabis use: the US experience. World Psychiatry. 2020;19(2):179-186. doi:10.1002/wps.20735 32394566 PMC7215066

[33] Imtiaz S, Nigatu YT, Ali F, Douglas L, Hamilton HA, Rehm J, . Cannabis legalization and cannabis use, daily cannabis use and cannabis-related problems among adults in Ontario, Canada (2001–2019). Drug Alcohol Depend. 2023;244:109765. doi:10.1016/j.drugalcdep.2023.109765 36652851

[34] Duff C, Erickson PG. Cannabis, risk and normalisation: evidence from a Canadian study of socially integrated, adult cannabis users. Health Risk Soc. 2014;16(3):210-226. doi:10.1080/13698575.2014.911823

[35] Wadsworth E, Driezen P, Chan G, Hall W, Hammond D. Perceived access to cannabis and ease of purchasing cannabis in retail stores in Canada immediately before and one year after legalization. Am J Drug Alcohol Abuse. 2022;48(2):195-205. doi:10.1080/00952990.2021.2003808 35157544

[36] Goodman S, Hammond D. Perceptions of the health risks of cannabis: estimates from national surveys in Canada and the United States, 2018-2019. Health Educ Res. 2022;37(2):61-78. doi:10.1093/her/cyac006 35311986 PMC8947787

[37] Craig P, Cooper C, Gunnell D, . Using natural experiments to evaluate population health interventions: new Medical Research Council guidance. J Epidemiol Community Health. 2012;66(12):1182-1186. doi:10.1136/jech-2011-200375 22577181 PMC3796763

