# Supplementary material for: Cannabis Use Frequency and Cannabis-Related Consequences in High-Risk Young Adults Across Cannabis Legalization
Source: JAMA Netw Open. 2023 Sep 27;6(9):e2336035. doi: 10.1001/jamanetworkopen.2023.36035 (PMC10534274; doi:10.1001/jamanetworkopen.2023.36035)
Supplement: Supplement 1. — eTable. Attrition Analysis eFigure 1. Distribution of Participation Across 7 Study Waves eFigure 2. Prelegalization B-MACQ and CUDIT Summaries by Cannabis Use Category [file jamanetwopen-e2336035-s001.pdf]

Supplemental Online Content

Doggett A, Belisario K, McDonald AJ, Ferro MA, Murphy JG, MacKillop J. Cannabis use frequency and cannabis-related consequences in high-risk young adults across cannabis legalization. *JAMA Netw Open*. 2023;6(9):e2336035.
doi:10.1001/jamanetworkopen.2023.36035

eFigure 1. Distribution of Participation Across 7 Study Waves
eTable. Attrition Analysis
eFigure 2. Prelegalization B-MACQ and CUDIT Summaries by Cannabis Use Category

This supplemental material has been provided by the authors to give readers additional information about their work.

© 2023 Doggett A et al. *JAMA Network Open.*

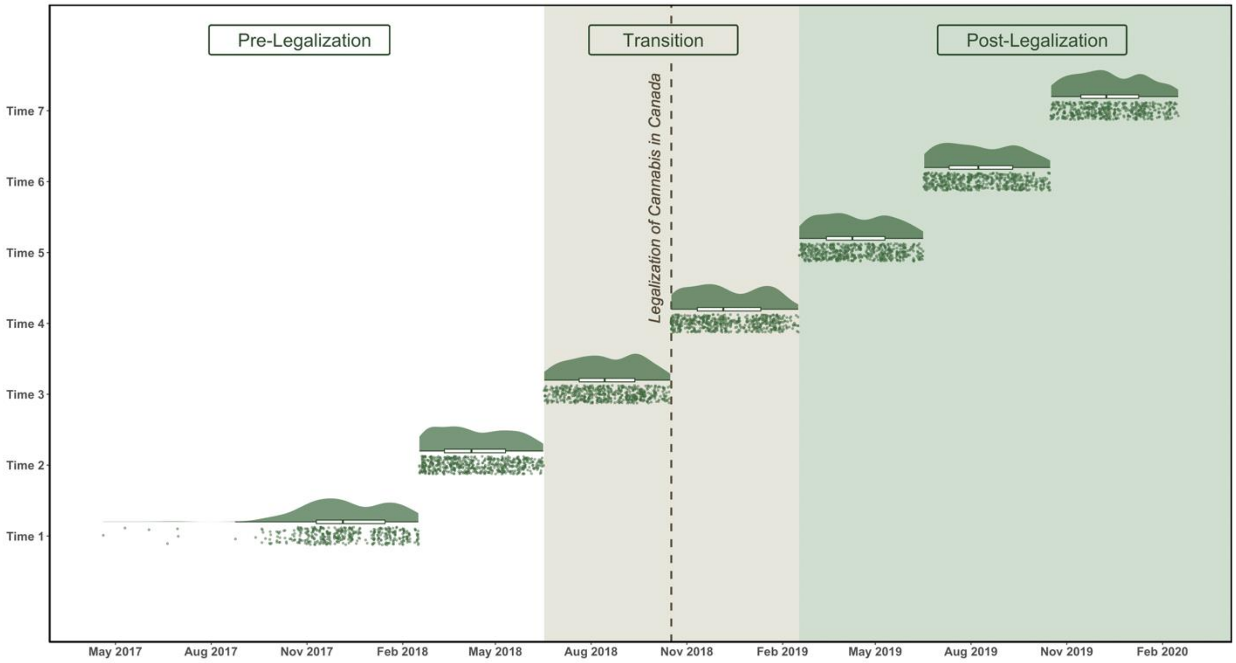

eFigure 1. Distribution of Participation Across 7 Study Waves

© 2023 Doggett A et al. *JAMA Network Open.*

eTable. Attrition Analysis

Baseline Characteristics	Mean (SD)		p-value[1]
	**Included (N = 619)**	**Excluded (N = 107)**	
Female Sex, N (%)	345 (55.7%)	37 (34.6%)	<0.001
Non-White Ethnicity, N (%)	191 (30.9%)	26 (24.3%)	0.21
Age	21.43 (1.19)	21.41 (1.16)	0.86
Cannabis ASSIST[2]	1.08 (0.05)	1.49 (0.14)	0.006
B-MACQ[3] Score	3.76 (0.22)	5.56 (0.64)	0.009

[1]Chi-square test of independence
[2]ASSIST: Alcohol, Smoking, Substance Involvement Screening Test (ASSIST). Response options are "none," "monthly," "weekly," "daily," or "multiple times daily."
[3]B-MACQ: Brief Marijuana Consequences Questionnaire.

© 2023 Doggett A et al. *JAMA Network Open.*

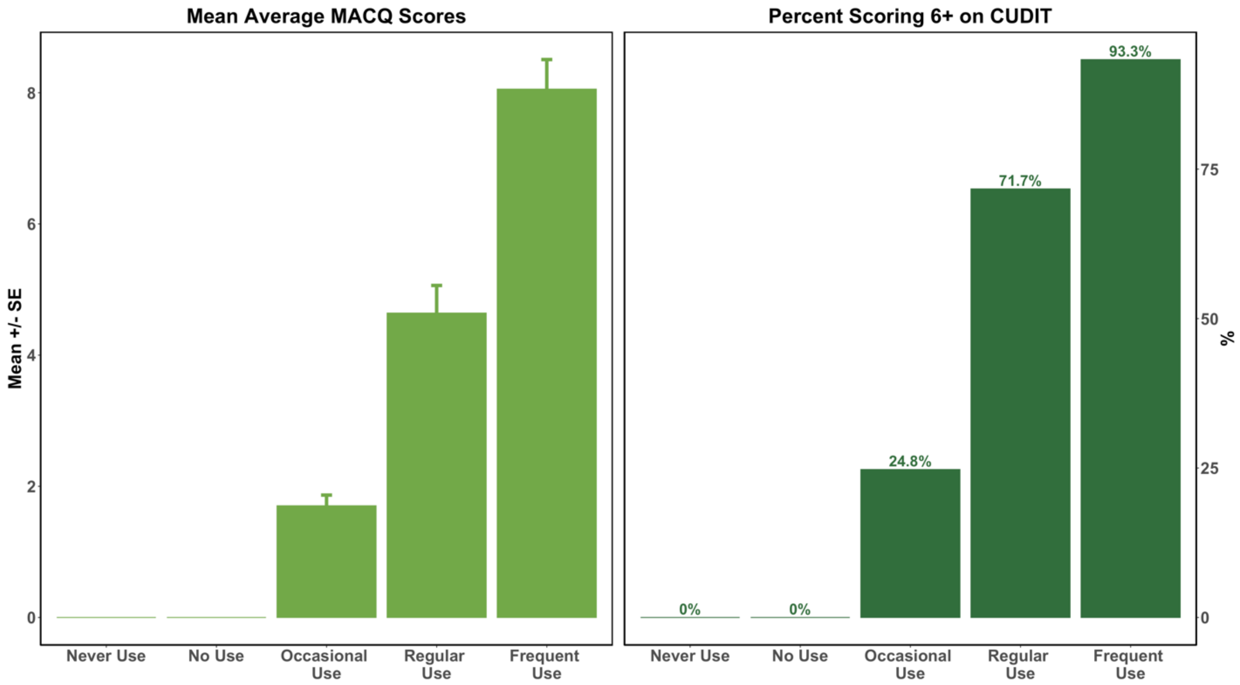

eFigure 2. Prelegalization B-MACQ and CUDIT Summaries by Cannabis Use Category
CUDIT: Cannabis Use Disorders Identification Test
B-MACQ: Brief Marijuana Consequences Questionnaire

© 2023 Doggett A et al. *JAMA Network Open.*
