# Supplementary material for: Cannabis Use Frequency and Cannabis-Related Consequences in High-Risk Young Adults Across Cannabis Legalization
Source: JAMA Netw Open. 2023 Sep 27;6(9):e2336035. doi: 10.1001/jamanetworkopen.2023.36035 (PMC10534274; doi:10.1001/jamanetworkopen.2023.36035)
Supplement: Supplement 2. — Data Sharing Statement [file jamanetwopen-e2336035-s002.pdf]

Data Sharing Statement

Doggett. Cannabis Use Frequency and Cannabis-Related Consequences in High-Risk Young Adults Across Cannabis Legalization. *JAMA Netw Open*. Published September 27, 2023. doi:10.1001/jamanetworkopen.2023.36035

Data
Data available: No
